# Autophagy is a pro-survival adaptive response to heat shock in bovine cumulus-oocyte complexes

**DOI:** 10.1038/s41598-020-69939-3

**Published:** 2020-08-13

**Authors:** Lais B. Latorraca, Weber B. Feitosa, Camila Mariano, Marcelo T. Moura, Patrícia K. Fontes, Marcelo F. G. Nogueira, Fabíola F. Paula-Lopes

**Affiliations:** 1 Department of Pharmacology, Institute of Bioscience, São Paulo State University (UNESP), District of Rubião Junior S/N, Botucatu, São Paulo 18618970 Brazil; 2 Department of Biological Sciences, Federal University of São Paulo, Diadema, São Paulo 09972270 Brazil; 3 Department of Biological Sciences, School of Sciences and Languages, UNESP, Assis, São Paulo Brazil

**Keywords:** Macroautophagy, Embryology

## Abstract

Autophagy is a physiological mechanism that can be activated under stress conditions. However, the role of autophagy during oocyte maturation has been poorly investigated. Therefore, this study characterized the role of autophagy on developmental competence and gene expression of bovine oocytes exposed to heat shock (HS). Cumulus-oocyte-complexes (COCs) were matured at Control (38.5 °C) and HS (41 °C) temperatures in the presence of 0 and 10 mM 3-methyladenine (3MA; autophagy inhibitor). Western blotting analysis revealed that HS increased autophagy marker LC3-II/LC3-I ratio in oocytes. However, there was no effect of temperature for oocytes matured with 3MA. On cumulus cells, 3MA reduced LC3-II/LC3-I ratio regardless of temperature. Inhibition of autophagy during IVM of heat-shocked oocytes (3MA-41 °C) reduced cleavage and blastocyst rates compared to standard in vitro matured heat-shocked oocytes (IVM-41 °C). Therefore, the magnitude of HS detrimental effects was greater in the presence of autophagy inhibitor. Oocyte maturation under 3MA-41 °C reduced mRNA abundance for genes related to energy metabolism (*MTIF3*), heat shock response (*HSF1*), and oocyte maturation (*HAS2* and *GREM1*). In conclusion, autophagy is a stress response induced on heat shocked oocytes. Inhibition of autophagy modulated key functional processes rendering the oocyte more susceptible to the deleterious effects of heat shock.

## Introduction

Autophagy is a programmed lysosomal process that degrades damaged or unnecessary cellular proteins, lipids, DNA, RNA, and organelles for recycling amino acids, fatty acids, and nucleosides to act as cellular building blocks for anabolic processes^1–3^. This event is involved in development, differentiation, immunity, aging, and cell death^2^. The autophagy pathway can be triggered by different stress conditions in order to restore homeostasis as a pro-survival adaptive response to stress. For example, autophagy can be induced by diseases^4–6^ and environmental factors such as nutrient deprivation^7,8^, high temperature^9–11^, oxidative stress^12,13^, hypoxia^14,15^, and toxins^16–18^.

There are three autophagy types described in mammalian cells: chaperone-mediated autophagy, macroautophagy, and microautophagy^1^. In the chaperone-mediated autophagy, heat shock cognate 70 complex (Hsc70 complex) directs abnormal proteins that have KFERQ-like motifs exposed to lysosome-mediated degradation. On the other hand, during microautophagy the lysosome membrane invaginates, thus capturing a small portion of the cytoplasm to be degraded^2^. Macroautophagy is the best-understood type of autophagy, which is mediated by double-membrane vesicles that transport abnormal proteins and organelles through the cytoplasm (i.e., autophagosomes). The autophagosome membrane fuses to lysosomes to breakdown the transported material^2^.

During embryonic development, autophagy is first induced after fertilization to clear sperm-born material that is not necessary for further embryogenesis^19–21^. In turn, autophagy is also associated with abundance of maternal mRNA, such as *C-mos* and *Cyclin B*^22,23^, and it seems to have an important role before mouse and porcine embryo genome activation^23,24^. Inhibition or induction of autophagy with 3-methyladenine (3MA) and rapamycin, respectively, during culture of porcine embryos from one-cell to the four-cell stage or one-cell to the blastocyst stage affected embryo developmental potential, blastocyst cell number, and apoptosis index. However, the same treatments had no effect from the four-cell to the blastocyst stage^23^, demonstrating that autophagy is only required for early stages of preimplantation development.

Even though induction of autophagy has been shown to play a role after oocyte activation and during preimplantation development^21^, little has been demonstrated on the role of autophagy on oocyte maturation^25^. The autophagy-specific marker microtubule-associated protein 1 light chain-II (LC3-II) known to associate with the autophagosomal membrane was increased in porcine oocytes matured in vitro^26^. While induction of autophagy during porcine in vitro maturation (IVM) improved oocyte maturation, fertilization, blastocyst development, and quality^27^, autophagy inhibition disrupted oocyte DNA and mitochondria, increased reactive oxygen species production, apoptosis, and reduced embryonic development^28^.

There are several environmental stressors that can induce autophagy during oocyte maturation^16–18^. A well-known stressor for oocytes is elevated temperature, to which bovine oocytes are often exposed leading to reduced fertility^29,30^. The deleterious effects of elevated temperature on bovine oocytes has been well characterized^30–36^. However, the molecular mechanisms involved in these processes are not fully understood. As the oocyte has limited ability to respond to stress, understanding the oocyte survival signaling pathways would provide new alternatives to overcome the deleterious effects of heat stress. Therefore, the hypothesis of this study was that autophagy is a stress response activated during maturation of heat-shocked oocytes in order to restore homeostasis and promote survival. The objective was to determine whether autophagy pathway is induced by heat shock and the role of autophagy on developmental competence and gene expression of bovine oocytes exposed to heat shock during IVM.

## Materials and methods

### Materials

Unless otherwise stated, all chemicals were purchased from Sigma-Aldrich (St. Louis, MO, USA). Tissue Culture Medium-199 (TCM-199 containing l-glutamine and phenol red) and fetal bovine serum (FBS) were purchased from GIBCO (Grand Island, NY, USA). Folltropin–V (Follicle-stimulating hormone; FSH) was purchased from Bioniche Animal Health Canada Inc. (Bellevile, Ontario, Canada) and Chorulon (Human chorionic gonadotrophin; hCG) from Intervet Schering Plough (Roseland, NJ, USA). EmbryoMax (KSOM Powdered Media Kit #MR-020P-5F) was purchased from MilliPore (Livingston, Fleming Road, UK). The autophagy inhibitor 3-Methyladenine (3MA; #sc-205596) was purchased from Santa Cruz Biotechnology (Dallas, TX, USA). The primary antibody [LC3A/B (D3U4C) rabbit monoclonal antibody—#12741] and secondary antibody (anti-rabbit IgG – HRP-linked antibody—#7074) were purchased from Cell Signaling Technology (Danvers, 3 Trash Lane, MA). Frozen semen was purchased from CRV Lagoa (Sertãozinho, São Paulo, Brazil).

### Experimental design

#### Experiment 1: Autophagy induction on bovine oocytes and cumulus cells exposed to heat shock during IVM

**Figure 1:**
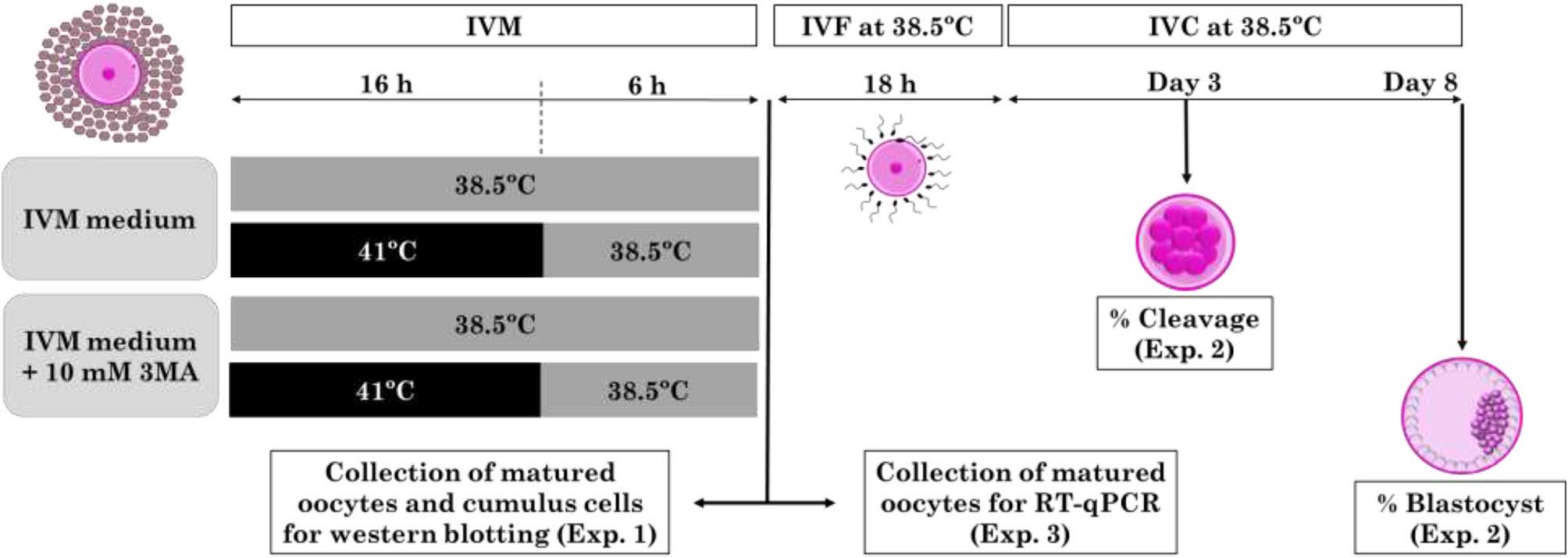
Schematic representation of the experimental design. COCs underwent IVM at Control (38.5 °C for 22 h) and Heat Shock (41 °C for 16 h followed by 38.5 °C for 6 h) temperatures in the presence of 0 or 10 mM 3MA. After 22 h IVM, COCs were either denuded and processed for western blotting and RT-qPCR analysis or submitted to IVF for 18 h at 38.5°C, followed by IVC for 8 days. Embryonic development was recorded on Days 3 (cleavage rate, %) and 8 (blastocyst rate, %) after IVF. IVM, in vitro maturation; IVF, in vitro fertilization; IVC, in vitro culture; 3MA, 3-Methyladenine. The figure was adapted from Master’s Thesis^83^.

The objective of this study was to determine autophagy activation and efficiency of the autophagy inhibitor 3-methyladenine (3MA) in cumulus-oocyte complexes (COCs) exposed to heat shock during IVM. 3MA inhibits the positive regulator of autophagy known as class III phosphatidylinositol-3-kinase (PI3K). This experiment was arranged in a 2 × 2 factorial design to determine the effect of temperature (38.5 or 41 °C) and autophagy inhibitor (0 or 10 mM 3MA) during IVM on autophagy activity by measuring LC3 protein level (autophagy marker). COCs were distributed on the following experimental groups: Control (38.5 °C for 22 h) and Heat Shock (41 °C for 16 h followed by 38.5 °C for 6 h^36,37^), in the presence of 0 or 10 mM 3MA during IVM^38^. After IVM, COCs were denuded so that denuded oocytes and respective cumulus cells were processed for western blotting analysis to determine autophagy activity by LC3-I and LC3-II level. This experiment was replicated 3 times using 60 oocytes/treatment/replicate and cumulus cells of 60–70 COCs/treatment/replicate (Fig. 1).

#### Experiment 2: The role of autophagy on developmental competence of heat-shocked bovine oocytes

The objective was to determine the role of autophagy on developmental potential of bovine oocytes exposed to heat shock during IVM. This experiment was arranged in a 2 × 2 factorial design as described for experiment 1. COCs were distributed on the following experimental groups: Control (38.5 °C for 22 h) and Heat Shock (41 °C for 16 h followed by 38.5 °C for 6 h) in the presence of 0 or 10 mM 3MA during IVM. After 22 h IVM, COCs were subjected to in vitro fertilization (IVF) and in vitro culture (IVC) as described below. Cleavage rate was determined at day 3 and the percentage of oocytes and cleaved embryos that reached the blastocyst stage was determined on day 8 post-IVF, respectively. This experiment was replicated 5 times using 30 COCs/treatment/replicate (Fig. 1).

#### Experiment 3: The effect of autophagy inhibition on gene expression of bovine oocytes exposed to heat shock

The objective was to determine the effect of autophagy on mRNA abundance of bovine oocytes submitted to heat shock during IVM. This experiment was arranged in a 2 × 2 factorial design and COCs were randomly distributed on the same experimental groups as described before. After IVM, pools of 30 oocytes were denuded, processed and stored at – 80 °C until RT-qPCR analysis using Biomark HD System. This experiment was replicated 5 times using 30 oocytes/treatment/replicate (Fig. 1).

## Methods

### In vitro maturation

Bovine ovaries (crossbred *Bos taurus indicus*) were collected at slaughterhouse and transported within 2 h to the laboratory in sterile saline [0.9% (w/v) NaCl containing 100 U/mL penicillin-G and 100 µg/mL streptomycin] at 37 °C. COCs were collected by slicing 2–8 mm follicles into Oocyte Collection Medium [Tissue Culture Medium-199 (TCM-199) containing L-glutamine and phenol red supplemented with 2.2 mg/mL sodium bicarbonate, 1% (v/v) fetal bovine serum (FBS) containing 2 U/mL heparin, 0.01 µg/mL streptomycin, 0.01 U/mL penicillin-G]. Grade I-III COCs^39^ were selected and washed once on pre-IVM medium [TCM-199 HEPES supplemented with 10% (v/v) FBS, 50 μg/mL gentamicin and 0.2 mM sodium pyruvate]. Groups of 10 COCs were placed on 50 µL drops of IVM medium [TCM-199-Bicarbonate containing 10% (v/v) FBS, 50 μg/mL gentamicin, 0.2 mM sodium pyruvate, 10 μg/mL FSH, 10 μg/mL hCG, and 1 μg/mL 17-β estradiol] in the presence of 0 or 10 mM 3-MA. IVM drops were covered with mineral oil at control (38.5 °C for 22 h under 5% CO_2_) or heat shock (41 °C for 16 h under 7% CO_2_ followed by 38.5 °C for 6 h under 5% CO_2_)^40,41^ temperatures in humidified air.

### Western blotting analysis

After IVM COCs were transferred to microcentrifuge tubes, vortexed in pre-warmed phosphate buffered saline (10 mM PBS) containing 0.1% (w/v) hyaluronidase for 5 min. Denuded oocytes were washed three times in 10 mM PBS containing 1 mg/mL PVP (polyvinylpyrrolidone) and suspended in 15 μL of buffer solution [1.5 M Tris–HCl pH 6.8 containing 50% (v/v) glycerol, 25% (v/v) β-mercaptoethanol, 10% (v/v) SDS, and 0.05% (v/v) bromophenol blue], and 15 μL of PBS-PVP, boiled at 90 °C for 5 min and stored at -80 °C. After vortexing, the remaining cumulus cells were washed in 1 mL PBS at 5,000 × g for 5 min, followed by a second wash in 500 μL PBS at 5,000 × g for 5 min. The pellet of cumulus cells was subjected to 5 cycles of thermal shock (1 min in liquid nitrogen followed by 1 min at 37 °C), resuspended in 15 μL of PBS-PVP and 15 μL of buffer solution, boiled at 90 °C for 5 min and stored at – 80 °C. Proteins were separated by 15% (w/v) sodium dodecyl sulfate–polyacrylamide gel electrophoresis (SDS-PAGE) gel at 60 mA. Gels were transferred to a polyvinylidenedifluoride membrane at 15 V for 1 h at room temperature (RT). Membranes were then incubated in blocking buffer [5% (w/v) skim powdered milk diluted in Tris (TBS) saline with 0.1% (v/v) Tween-20—(TBS-T)] for 1 h at RT. After blocking, membranes were washed 3 times in washing buffer [TBS-T] at RT and incubated with primary rabbit monoclonal antibody anti-LC3—I and II [1:1000]^26,42^ diluted in TBS-T containing 5% bovine serum albumin (BSA) at 4 °C overnight. Membranes were washed in TBS-T, incubated with secondary antibody [1:2000] anti-rabbit IgG diluted in TBS-T containing 5% BSA for 1 h at RT, washed 3 times, and incubated with chemiluminescent reagent (SuperSignal West Pico PLUS Chemiluminescent Substrate, 1:1) for 1 min at RT. Chemiluminescence was captured by Alliance LD4 Gel Documentation System (UVITEC Cambridge). The proteins LC3-I (16 kDa) and II (14 kDa) were identified according to molecular weight. Protein abundance was analyzed by UVIBANDMAX software, version 15.06a, through band size and intensity.

### In vitro fertilization and culture

After 22 h IVM, COCs were washed on pre-IVF medium (TCM-199 HEPES supplemented with 3 mg/mL BSA fraction V, 50 µg/mL gentamicin, and 0.2 mM sodium pyruvate) and on IVF medium [modified Tyrode's albumin-lactate-pyruvate (TALP) medium^43^ containing 6 mg/mL essentially fatty acid free (EFAF)-BSA, 50 µg/mL gentamicin, 0.2 mM sodium pyruvate, 100 µg/mL heparin, and 41.66 µL/mL PHE (penicillamine 2.7 µg/mL, hypotaurine 1 µg/mL, epinephrine 0.33 µg/mL in 0.9% (w/v) NaCl]. Groups of 10 COCs were placed in 90 µL drop of IVF medium covered with mineral oil at 38.5°C and 5% CO_2_ in humidified air. Frozen-thawed straws from two different bulls (*Bos taurus indicus*) were used for each replicate. Viable spermatozoa were purified by centrifugation (9,000 × g for 5 min) on Percoll gradient (45% and 90%) followed by centrifugation (9,000 × g for 2.5 min) on SP-TALP medium (TALP medium containing 6 mg/mL EFAF-BSA, 50 µg/mL gentamicin, and 0.2 mM sodium pyruvate). COCs were inseminated with 1 × 10^6^ sperm/mL at 38.5 °C and 5% CO_2_ in humidified air. After 18 h IVF, presumptive zygotes were denuded by 5 min vortexing in pre-IVF medium and washed three times in modified potassium simplex optimization medium (KSOM medium – Embryomax) supplemented with 10% (v/v) FBS, 1.25 µg/mL gentamicin, and 4 µl/mL non-essential amino acids (KSOMaaFBS). Groups of 25–30 presumptive zygotes were placed in 50 µL drops of KSOMaaFBS covered with mineral oil at 38.5°C and 5% CO_2_ in humidified air for 8 days. The percentage of oocytes that cleaved was determined at day 3 after IVF and the percentage of oocytes and cleaved embryos that reached the blastocyst stage was determined at day 8 after IVF.

### cDNA synthesis and gene expression analysis

Total RNA was extracted from pools of 30 denuded oocytes (n = 5 pools/treatment/replicate) using Qiagen RNeasy Micro Kit (Qiagen Inc., Valencia, CA, USA) following the manufacturer’s instructions. DNAse treatment was performed in all samples during RNA isolation according to the manufacturer’s instructions. The cDNA synthesis was performed using all the RNA with the High Capacity Reverse Transcription kits (Thermo Fisher Scientific, Waltham, MA, USA), following the manufacturer’s instructions.

Gene expression analysis of bovine oocytes was performed using Applied BiosystemsTaqMan Assays, specific for *Bos taurus* species as previously described by Razza et al.^44,45^. A total of 96 candidate genes were analyzed (Table 1). Prior to qPCR thermal cycling, each cDNA sample was submitted to sequence-specific preamplification process as follows: 1.25 µL assay mix (Taqman Assay was pooled to a final concentration of 0.2 × for each of the 96 assays), 2.5 µL TaqManPreAmp Master Mix (Applied Biosystems, #4391128) and 1.25 µL cDNA. The reactions were activated at 95 °C for 10 min followed by denaturing at 95 °C for 15 s, annealing and amplification at 60 °C for 4 min for 14 cycles. Preamplified products were diluted fivefold prior to RT-qPCR analysis. The preamplification process is a required step for analysis in the Biomark HD system due to the nanoliter scale of qPCR reactions. A recent study using the same microfluidic platform demonstrated that preamplification step performed on 96.96 Biomark HD Array for bovine oocytes was uniform and reliable^45^.

**Table 1:** Genes evaluated by RT-qPCR using Applied BiosystemsTaqMan Assay^83^.

Gene ID	Initials	Full name
Bt03213473_m1	AGTR1	Angiotensin II receptor type 1
AIY9Z3D	AGTR2	Angiotensin II receptor type 2
Bt03271014_m1	AREG/LOC538751	Amphiregulin
Bt03213774_m1	ARO	Aromatase
Bt03221057_m1	ATF4	Activating transcription factor 4
Bt03210836_g1	ATP5L	ATP synthase, H + transporting, mitochondrial Fo complex subunit E
Bt03275798_m1	AUH	AU RNA binding protein
Bt03251628_m1	BAX	BCL2-Associated X Protein
Bt03211777_g1	BCL2	B-cell CLL/lymphoma 2
Bt04298952_m1	BID	BH3 interacting domain death agonist
Bt03241255_m1	BMP15	Bone morphogenetic protein 15
Bt03286494_u1	CASP3	caspase 3, apoptosis-related cysteine peptidase
Bt03250954_g1	CASP9	Caspase 9, apoptosis-related cysteine peptidase
Bt04282453_m1	CAT	Catalase
Bt03228713_m1	CCND2	Cyclin D2
Bt03249250_m1	CDCA8	Cell division cycle associated 8
Bt03257041_g1	CDK6	Cyclin-dependent kinase 6
Bt04311264_m1	DDIT3	DNA-damage-inducible transcript 3
Bt03251320_g1	DICER1	Dicer 1, Ribonuclease III
Bt03217754_m1	DNMT1	DNA (Cytosine-5-)-Methyltransferase 1
Bt03224737_m1	DNMT3A	DNA (Cytosine-5-)-Methyltransferase 3A
Bt01027164_m1	DNMT3B	DNA (Cytosine-5-)-Methyltransferase 3B
Bt03259810_m1	EGFR	Epidermal growth factor—receptor
AJT96D7	FOXO3	Forkhead box O3
Bt03649334_s1	FSHR	Follicle-stimulating hormone receptor
Bt03212674_m1	G6PD	Glucose 6 phosphate desidrogenase
Bt03649181_m1	GADD45A	Growth arrest and DNA-damage-inducible, alpha
Bt03225650_m1	GAPDH	Glyceraldehyde-3-phosphate dehydrogenase
Bt03210912_g1	GDF9	Growth differentiation factor 9
Bt03223996_m1	GFPT2	Glutamine-fructose-6-phosphate transaminase 2
Bt03250351_m1	GLRX2	Glutaredoxin 2
Bt03229700_m1	GPAM	Glycerol-3-phosphate acyltransferase, mitochondrial
Bt03210381_m1	GPX1	Glutathione peroxidase 1
Bt03259217_g1	GPX4	Glutathione peroxidase 4
Bt03259611_m1	GREM1	Gremlin 1
Bt03255355_m1	GSK3A	Glycogen synthase kinase 3 alpha
Bt03273695_m1	H1FOO	H1 histone family, member O, oocyte-specific
Bt03228652_g1	HAS2	Hyaluronan synthase 2
Bt03212695_g1	HDAC2	Histone deacetylase 2
Bt03244871_m1	HIF1A	Hypoxia inducible factor 1 (transcription factor)
Bt03259341_m1	HSF1	Heat shock transcription factor 1
Bt03249686_m1	HSP90AA1	Heat Shock Protein 90kda Alpha
Bt03218068_g1	HSPA1A	Heat shock 70 kDa protein 1A
Bt03292670_g1	HSPA5	Glucose-regulated protein, 78 kDa
Bt03244880_m1	HSPD1	Heat shock 60 kDa protein 1A
Bt04301470_g1	IGF1R	Insulin-like growth factor 1 receptor
Bt03649217_m1	IGF2	Insulin-like growth factor 2
Bt03259224_m1	IGFBP2	Insulin-like growth factor binding protein 2
Bt01040719_m1	IGFBP4	Insulin-like growth factor binding protein 4
Bt03259500_m1	KEAP1	Kelch-like ECH-associated protein 1
Bt03817661_m1	LHCGR	Luteinizing hormone/choriogonadotropin receptor
Bt03213974_m1	MAPK1	Mitogen-activated protein kinase
Bt03216718_g1	MTIF3	Mitochondrial translational initiation factor 3
Bt03231844_m1	NANOG	Nanoghomeobox
Bt03220541_m1	NFE2L2	Nuclear factor (erythroid-derived 2)-like 2
Bt03251880_m1	NFKB2	Nuclear factor of kappa light polypeptide gene enhancer in b-cells 2
Bt03272789_g1	NOS2	Nitric oxide synthase 2, inducible
Bt03249597_m1	NOS3	Nitric oxide synthase 3
Bt03217679_m1	NPPA	Natriuretic peptide A (ANP)
Bt03223175_g1	NPPB	Natriuretic peptide B (BNP)
Bt04301375_g1	NPPC	Natriuretic peptide C (CNP)
Bt03212844_m1	NPR1	Natriuretic peptide receptor 1
Bt04297034_g1	NPR2	Natriuretic peptide receptor 2
Bt04316732_m1	NPR3	Natriuretic peptide receptor 3
Bt03212867_m1	NR1H3	Nuclear receptor subfamily 1 group H member 3
Bt03218363_m1	OOSP1	Oocyte-secreted protein 1
Bt03233533_g1	PA2G4	Proliferation-associated 2G4
Bt03211241_g1	PAF1	RNA polymerase ii associated factor
Bt03239371_g1	PDE5A	Phosphodiesterase 5A, cGMP-specific
Bt03214261_m1	PFKP	Phosphofructokinase
Bt04316551_m1	PGK1	Phosphoglycerate kinase 1
Bt03225854_mH	PNPLA2	Patatin-like phospholipase domain containing 2
Bt03234129_g1	POU5F1	POU class 5 homeobox 1 (OCT4)
Bt03223846_g1	PPARA	Peroxisome proliferator-activated receptor alpha
Bt03220821_m1	PPARG	Peroxisome proliferator-activated receptor gamma
Bt03217547_m1	PPARGC1A	Peroxisome proliferator-activated receptor gamma, coactivator 1 alpha
Bt01016720_m1	PPIA	Peptidylprolylisomerase A
Bt03224617_g1	PRDX1	Peroxiredoxin-1
Bt03223684_m1	PRDX3	Peroxiredoxin 3
Bt03214402_m1	PTGS2/COX2	Prostaglandin-endoperoxide synthase 2
Bt03214489_m1	PTX3	Pentraxin 3, long
Bt03249011_m1	REST	RE1-silencing transcription factor
Bt03278318_s1	RGS2	Regulator of G-protein signaling 2
Bt03246656_g1	RPL15	Ribosomal protein L15
Bt03288449_g1	SOD1	Superoxide dismutase 1, soluble
Bt03215423_g1	SOD2	Superoxide dismutase 2, mitochondrial
Bt03244551_m1	SOX2	SRY (sex determining region Y)-box 2
Bt03278318_s1	SREBF1	Sterol regulatory element binding transcription f1
Bt03276370_m1	SREBF2	Sterol regulatory element binding transcription f2
Bt04283467_m1	STAT3	Signal transducer and activator of transcription 3
Bt03259871_g1	TFAM	Transcription factor A, mitochondrial
Bt03260078_m1	TNFAIP6	Tumor necrosis factor, alpha-induced protein 6
Bt03210223_m1	TNFRSF21	Tumor necrosis factor receptor superfamily member 21
Bt03250597_m1	TP53	Tumor protein p53
Bt03223213_m1	VCAN	Versican
Bt03217633_m1	XBP1	X-box binding protein 1

For gene expression analysis, the sample solution consisted of 2.25 µL cDNA (preamplified products), 2.5 µL of TaqMan Universal PCR Master Mix (2 ×, Applied Biosystems) and 0.25 µL of 20 × GE Sample Loading Reagent (Fluidigm); and the assay solution: 2.5 µL of 20 × TaqMan Gene Expression Assay (Applied Biosystems) and 2.5 µL of 2 × Assay Loading Reagent (Fluidigm). The 96.96 Dynamic Array Integrated Fluidic Circuits (Fluidigm) chip was used for data collection. After priming, the chip was loaded with 5 µL of each assay solution and 5 µL of each sample solution. The qPCR thermal cycling was performed in the United Biomark HD System (Fluidigm, South San Francisco, CA, USA) using TaqMan GE 96 × 96 Standard protocol. Briefly, the protocol consisted of one stage of Thermal Mix (50 °C for 2 min, 70 °C for 20 min and 25°C for 10 min) followed by a Hot Start stage (50 °C for 2 min and 95 °C for 10 min), followed by 40 cycles of denaturation (95 °C for 15 s), primer annealing and extension (60 °C for 60 s).

The RefFinder software (GeNorm and Normfinder) was used to determine the most stable genes among samples. The retrieved reference genes were peptidyl-prolylisomerase-A (*PPIA*) and ribosomal protein L15 (*RPL15*). Therefore, the data were normalized by the formula: ΔCq = (target gene Cq)—(Geometric Mean of *PPIA* and *RPL15* genes). The data were then transformed (twofold change-ΔCq) and the result was used for statistical analyses^46^.

### Statistical analysis

Assumptions for analysis of variance (ANOVA) (normally distributed data and homogeneity of variance) were determined by JMP, Version 11 (SAS Institute Inc., Cary, NC, 1989–2007). Logarithmic and square root transformation were applied to obtain normal distribution whenever required. Parametric data were analyzed by least-squares ANOVA using General Linear Models (GLM) procedure of SAS (SAS, 1989). Dependent variables were LC3-II/LC3-I ratio, percentage of cleaved embryos, percentage of oocytes and cleaved embryos that developed to the blastocyst stage and fold change of analyzed genes. Independent variables were temperature, autophagy inhibitor, and replicate. The statistical model considered all the main effects and all possible interactions. Differences between individual means were further analyzed by completing pair-wise comparisons (probability of difference analysis; SAS Institute, Inc.). Non-parametric data (*HSPA5*, *PFKP*, and *XBP1* mRNA) were analyzed by the Kruskal–Wallis test. Differences of P < 0.05 were considered significant.

## Results

### Experiment 1: Autophagy induction on bovine oocytes and cumulus cells exposed to heat shock during IVM

**Figure 2:**
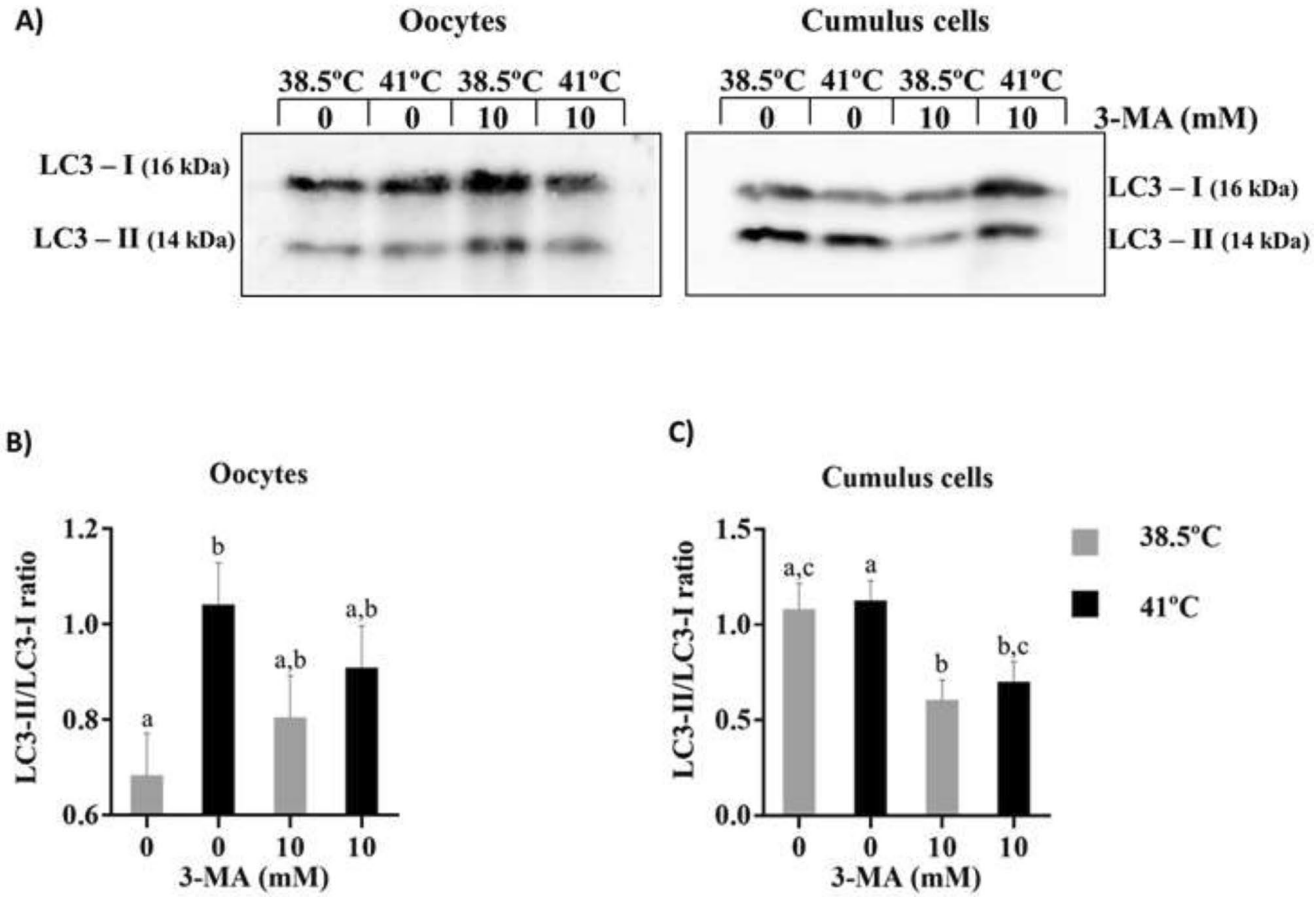
Induction of autophagy on COCs submitted to heat shock during IVM. Representative western blotting images of single cropped blots for oocytes and cumulus cells showing both types of LC3 protein (Full-length blots are presented in Supplementary Fig. S1 online) **(A)**. Quantification of LC3-II/LC3-I ratio on oocytes **(B)** and cumulus cells **(C)**. Results are least-squares means ± SEM. Different letters in each bar represent significant difference (P < 0.05).

The abundance of LC3-I and -II were differently detected in cumulus cells and oocytes, demonstrating cellular specificity to stress response during exposure to heat shock (Fig. 2 and Supplementary Data). The LC3-II/LC3-I ratio was used to evaluate autophagy induction, as LC3-I is conjugated with phosphatidylethanolamine (PE) to become LC3-II, which is part of the autophagosome membrane^28^. On oocytes, exposure to heat shock during IVM increased (P < 0.05) LC3-II/LC3-I ratio compared to oocytes at 38.5 °C, indicating the induction of autophagy by heat shock. Autophagy inhibition with 3MA did not change oocyte LC3-II/LC3-I ratio regardless of temperature (Fig. 2B). On cumulus cells, there was no effect of temperature on LC3-II/LC3-I ratio. However, addition of 3MA at 38.5 and 41 °C reduced (P < 0.05) LC3-II/LC3-I ratio, demonstrating that the drug was able to decrease autophagy activity in cumulus cells (Fig. 2C).

### Experiment 2: The role of autophagy on developmental competence of heat-shocked bovine oocytes

**Figure 3:**
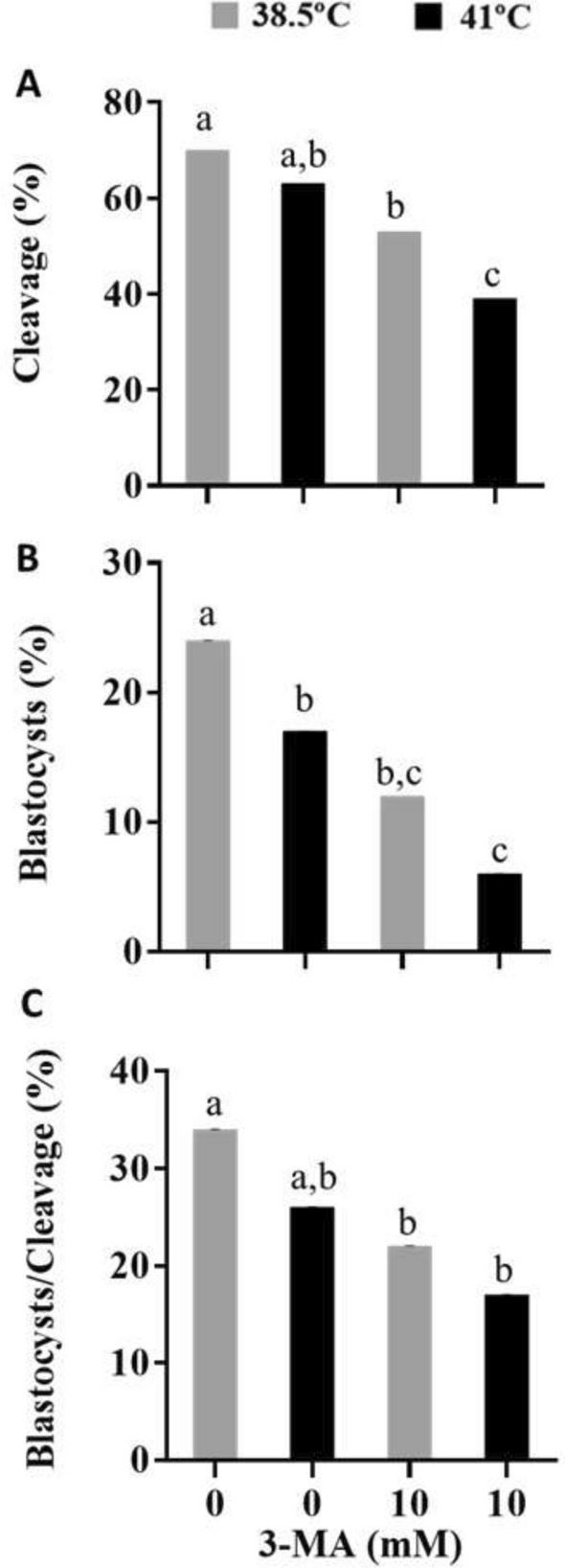
The impact of autophagy inhibition during IVM of heat-shocked bovine oocytes on the proportion of oocytes that cleaved **(A)** and developed to the blastocyst stage **(B)**, and the proportion of cleaved embryos that developed to blastocyst **(C)**. Results are least-squares means ± SEM. Different letters in each bar represent significant difference (P < 0.05). The figure was adapted from Master’s Thesis^83^.

In the absence of 3MA, heat shock (IVM-41 °C) did not affect cleavage rate (Fig. 3A). However, inhibition of autophagy reduced cleavage rate (P < 0.01) in heat-shocked oocytes (3MA-41 °C) when compared to all other groups (Fig. 3A). Moreover, the proportion of cleaved embryos was greater (P < 0.01) for oocytes matured in 0 mM 3MA than 10 mM 3MA at 38.5 °C (Fig. 3A). Bovine oocytes exposed to heat shock in the absence (IVM-41 °C; P < 0.05) or presence of 10 mM 3MA (3MA-41 °C; P < 0.01) reduced the proportion of oocytes that developed to the blastocyst stage at day 8 post-insemination compared with oocytes matured without 3MA at 38.5 °C (Fig. 3B). Addition of 10 mM 3MA at 38.5 and 41 °C reduced (P < 0.05) blastocyst rate compared to oocytes matured at the same temperature without 3MA, respectively. There was no effect of heat shock on the proportion of cleaved embryos that reached the blastocyst stage, regardless of 3MA (Fig. 3C). However, inhibition of autophagy reduced the proportion of cleaved embryos that developed to the blastocyst stage at 38.5 °C (P < 0.05) and 41 °C (P < 0.01) compared to oocytes matured in 0 mM 3MA at 38.5 °C (Fig. 3C).

Taken together, these results showed that autophagy activity during bovine oocyte maturation has a physiological role in early cleavage and development. Moreover, it has an important pro-survival effect in oocytes subjected to heat shock.

### Experiment 3: Inhibition of autophagy affects gene expression of bovine oocytes exposed to heat shock

**Figure 4:**
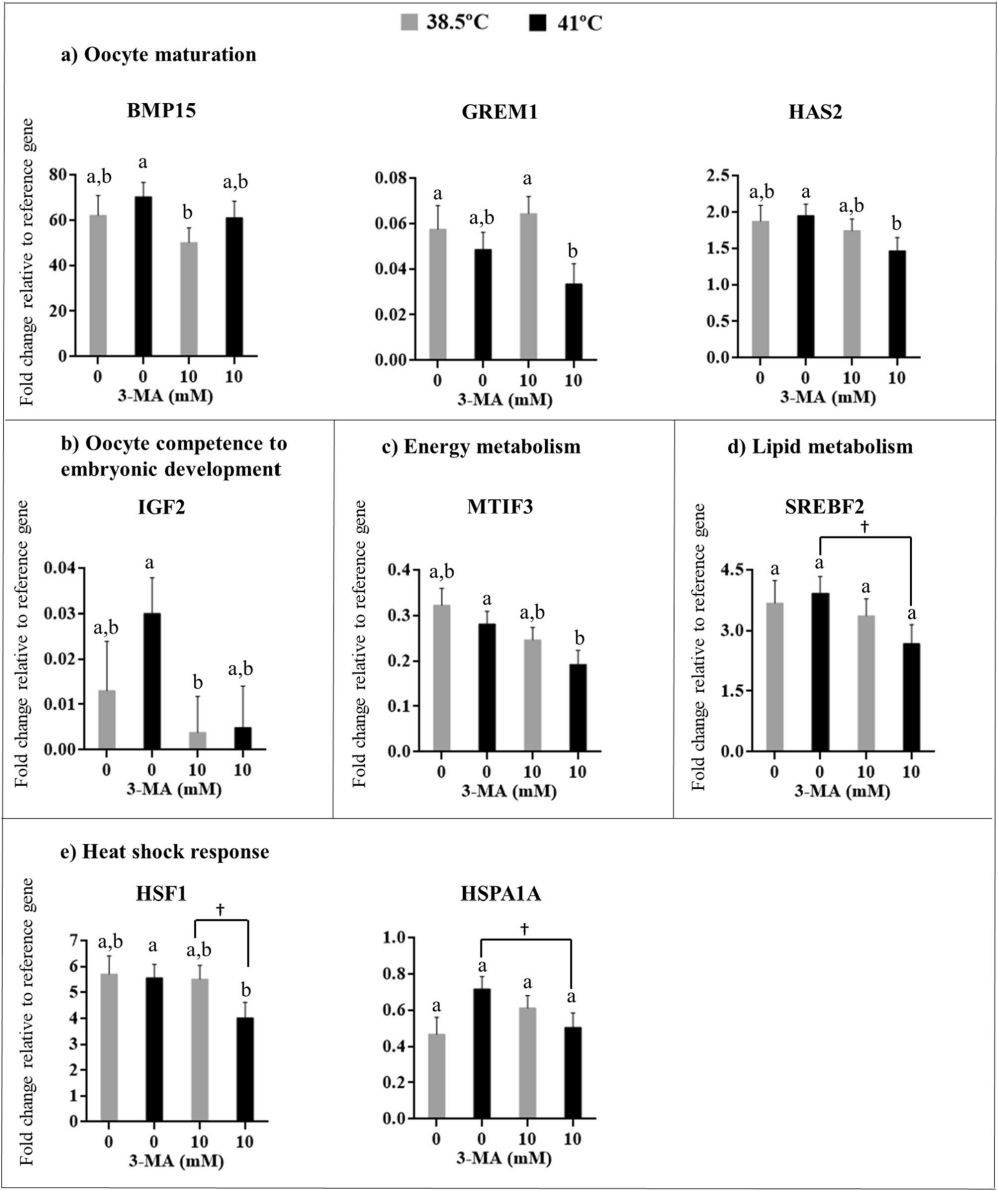
Effect of autophagy inhibition during IVM of heat-shocked bovine oocytes on mRNA abundance of differentially expressed genes according to the following functional categories: oocyte maturation **(A)**, oocyte competence to embryonic development **(B)**, energy metabolism **(C)**, lipid metabolism **(D)**, and heat shock response **(E)**. Results are least-squares means ± SEM. Different letters in each bar represent significant difference (P ≤ 0.05). ^†^P = 0.06. The figure was adapted from Master’s Thesis^83^.

Exposure of bovine oocytes to heat shock in the presence of 10 mM 3MA (3MA-41 °C) reduced transcript abundance of oocyte *HAS2* (P < 0.05; Fig. 4A), *MTIF3* (P < 0.05; Fig. 4C), and *HSF1* (P = 0.05; Fig. 4E) compared to heat-shocked oocytes in 0 mM 3MA (IVM-41 °C). There was a similar tendency for *SREBF2* (P = 0.06, Fig. 4D) and *HSPA1A* (P = 0.06, Fig. 4E). *GREM1* (P < 0.05; Fig. 4A) mRNA abundance were lower in heat-shocked oocytes matured in 10 mM 3MA (3MA-41 °C) than oocytes matured at 38.5 °C (3MA-38.5 °C). There was a tendency for similar pattern of expression for *HSF1* (P = 0.06). Inhibition of autophagy during oocyte maturation at 38.5 °C (3MA-38 °C) reduced *BMP15* (P < 0.05) and *IGF2* (P < 0.05) mRNA abundance in relation to heat shocked oocytes without 3MA (IVM-41 °C; Fig. 4A, B). String analysis of the significant genes revealed that *BMP15*, *HAS2*, and *GREM1* were frequently co-mentioned on publications (see Supplementary Fig. S2 online). There was no effect of temperature, autophagy inhibitor, and temperature *x* inhibitor interaction for all the other genes evaluated (see Supplementary Table S1 online). This reduced difference in oocyte mRNA abundance between groups was also evidenced in Heatmap analysis, where the majority of genes were similarly expressed in all groups (see Supplementary Fig. S3 online).

## Discussion

This study demonstrated that the bovine oocyte was able to undergo autophagy after exposure to adverse conditions. Autophagy acted as a pro-survival response during oocyte maturation as the developmental capacity of heat-shocked oocytes was markedly reduced in the presence of the autophagy inhibitor. The negative effect of autophagy inhibition also extended to gene expression. For example, mRNA abundance for genes related to oocyte maturation, energy metabolism and heat shock response were reduced in heat-shocked oocytes.

Activation of autophagy is usually studied through gene expression and protein abundance analysis of molecules involved in the autophagy pathway. The conversion of LC3-I to LC3-II is used as an autophagy marker, since LC3-II is necessary for autophagosome formation^28,47^. The present study provided evidence that oocyte autophagy was induced by heat shock during IVM as LC3-II/LC3-I ratio was increased on heat-shocked oocytes compared to control. However, heat-induced increase in autophagosome formation was not observed on cumulus cells. These results indicate a divergence between oocytes and cumulus cells response to stress condition^48,49^. It is well known that the ability of the oocyte to respond to stress is reduced during maturation due to limited transcriptional capacity^50^. The oocyte relies on translation of stored mRNA to activate pathways to restore homeostasis^51^. On the other hand, cumulus cells are somatic cells fully able to respond to stress conditions. Therefore, autophagy activation may play a greater role as a protection strategy for oocytes than cumulus cells.

Class III phosphatidylinositol-3-kinase (PI3K) is a positive regulator of autophagy, stimulating the initiation and maturation of autophagosome^52,53^ and 3MA acts inhibiting class III PI3K. Incubation of bovine COCs with 10 mM 3MA for 22 h did not reduce oocyte LC3-II/LC3-I ratio at the end of maturation period. However, a reduction in LC3-II/LC3-I ratio was clearly observed in cumulus cells that surrounded the oocytes. Therefore, the inhibitory effect of 3MA on COCs autophagy may be occurring indirectly through cumulus cells, impairing cleavage and blastocyst development. It is well known that cumulus cells are essential for oocyte survival and mediate several biological events regulating oocyte growth and maturation^54,55^. It has been shown that incubation of bovine oocytes with 5 mM 3MA also did not reduce LC3-II/LC3-I ratio at the end of 24 h maturation^56^. It is possible that the lack of 3MA-induced drop LC3-II/LC3-I ratio is due to the kinetics of LC3-II expression in the oocyte. It has been demonstrated that oocyte LC3-II protein increases during oocyte maturation, reaching a peak at the middle of maturation, and decreasing after that until the end of maturation period^26^.

Exposure of oocytes to heat shock did not affect cleavage rate, but reduced development to the blastocyst stage. At least part of the deleterious effects of heat shock on oocyte function is mediated by cellular and molecular damage to the oocyte. For example, heat shock reduced mitochondrial activity^36^, caused cytoskeleton disorganization^35,36^, protein denaturation, mRNA degradation as well as apoptosis^32,57–59^. In the present study, inhibition of autophagy prone the oocyte more susceptible to heat shock reducing the ability to cleave and reach the blastocyst stage. Therefore, it is likely that autophagy exerted a protective mechanism during oocyte maturation under stress condition. Lack of autophagy through class III PI3K inhibition may have blocked another protective mechanism, such as heat shock response, preventing oocyte recycling of damaged organelles or protein to restore homeostasis.

Moreover, this study demonstrated that autophagy was also required to standard in vitro maturation since cleavage and blastocyst development were reduced upon autophagy inhibition at physiological temperature. Similar results were observed with autophagy manipulation during porcine oocyte maturation^27,28^, corroborating with the hypothesis that recycling of cellular compounds is a strategy used by the oocyte for proper maturation.

The importance of autophagy on clearance of maternal and paternal mRNA after fertilization is already known^19–23^. However, the role of autophagy on mRNA abundance of bovine oocytes is not fully understood^60^. During maturation, the oocyte relies on mRNA stored during oocyte growth phase in order to adapt and support early embryonic development. Therefore, it is likely that autophagy regulates mRNA abundance depending on the environmental condition. Indeed, inhibition of the autophagy pathway in heat-shocked oocytes affected mRNA abundance of *HAS2*, *GREM1*, *MTIF3*, and *HSF1*. Likewise, such alteration on mRNA abundance exerted downstream effects modulating oocyte developmental competence in the absence of autophagy.

Class III PI3K inhibition in heat-shocked COCs reduced *HAS2* and *GREM1* mRNA abundance, indicating that the ability of the COCs to produce hyaluronidase and undergo cumulus cells expansion could be compromised. In addition to the report of heat shock reduction on cumulus cells expansion of bovine COCs matured at 41 °C^61^, there is also evidence that autophagy pathway is involved in the regulation of this event^62,63^. Inhibition of poly (ADP-ribose) polymerase enzymes (PARPs) during maturation of porcine oocytes lead to *HAS2* downregulation^62^. Therefore, autophagy inhibition might have reduced *HAS2* mRNA abundance via PARPs. It has also been shown that *GREM1* stimulates cumulus cell expansion and granulosa cell luteinization through bone morphogenic protein (BMP) regulation^64,65^. Consequently, reduction in *HAS2* and *GREM1* mRNA abundance in heat-shocked oocytes lacking the autophagic response could lead to poor oocyte maturation, reducing subsequent oocyte developmental ability.

Besides the effect on mRNA abundance of genes related to cumulus cells expansion, class III PI3K inhibition under heat shock also influenced energy balance during oocyte maturation. While *MTIF3* expression was not affected by heat shock, autophagy inhibition under high temperature reduced MTIF3 expression. This gene encodes a translation initiation factor involved in mitochondrial protein synthesis^66^ required for energy production by oxidative phosphorylation^67^. Thus, a deficiency of oxidative phosphorylation due to alteration on *MTIF3* expression may disrupt oocyte energy balance, impairing further embryonic development.

Heat-induced cellular pathways were also modulated in oocytes unable to activate the autophagic response, suggested by the reduced mRNA abundance of *HSF1* in heat-shocked oocytes matured with class III PI3K inhibitor. This gene is a major player of the heat shock response (HSR) to maintain cytoplasmic protein homeostasis, as the induction of heat shock proteins (HSPs) following cellular stress is a conserved response preventing aggregation of denatured or misfolded proteins^68^. Heat shock transcription factor 1 (HSF1) is the major transcriptional regulator responsible for the expression of HSPs and it is highly expressed in oocytes^69^. Both autophagy and HSR can be activated during heat shock as protein management systems^70^. In this case, the lack of autophagy activity could induce a selective translation of HSR genes to reduce the damage caused by exposure to heat shock.

Interestingly, class III PI3K inhibition during maturation of bovine oocytes under physiological temperature (38.5 °C) reduced *BMP15* and *IGF2* mRNA abundance compared with heat-shocked oocytes without the inhibitor. *BMP15* regulates differentiation of cumulus cells^71,72^. It seems like autophagy can regulate *BMP15* mRNA abundance since it was increased with autophagy activation^73^, and decreased with autophagy inhibition, as demonstrated here. On the other hand, *IGF2* is a growth factor identified on bovine oocytes^74^, and it improves bovine embryo production^75^, in addition to porcine oocyte nuclear maturation^76^. The alteration on both transcripts can be associated with the reduction of cleavage and blastocyst rate.

Several genes evaluated in this experiment were not affected by heat shock and/or autophagy inhibition. The resistance of heat-shocked bovine oocytes to changes in mRNA abundance during IVM has been demonstrated in previous studies^46,77^. For example, exposure of oocytes from Holstein and Nelore cows—breeds of contrasting heat tolerance—to 12 h heat shock during IVM had little effect on oocyte mRNA abundance investigated by large scale microarray^46^. Similar results were demonstrated by Payton et al.^78^. In contrast, germinal vesicle (GV) oocytes exposed to seasonal summer heat stress did not show resistance to modulate gene expression^78–80^. Oocyte DNA changes configuration during follicular development, demonstrated by different levels of chromatin condensation at GV stages^81^. The transcriptional and translational capacity of the oocyte is elevated from primordial to antral follicular stages, to store maternal mRNA for further oocyte and early embryonic development^51^. During either in vivo or in vitro oocyte maturation, the transcription rate is minimum as the chromatin condenses^77,82^. Therefore, the lack of change in gene expression observed for most of the genes studied is probably due to oocyte chromatin configuration during maturation.

In conclusion, this study provided novel evidence that autophagy is an adaptive response required for survival of heat-shocked oocytes, in addition to its importance during physiological maturation. It also revealed some potentially relevant mechanisms triggered by autophagy inhibition that lead to the deleterious effect of heat shock on oocyte developmental capacity.

## Supplementary information

Supplementary Information.

## Data Availability

The datasets generated and/or analyzed during the current study are available from the corresponding author on reasonable request.
