# Supplementary material for: Autophagy is a pro-survival adaptive response to heat shock in bovine cumulus-oocyte complexes
Source: Sci Rep. 2020 Aug 13;10:13711. doi: 10.1038/s41598-020-69939-3 (PMC7426922; doi:10.1038/s41598-020-69939-3)
Supplement: Supplementary file 1 — Supplementary Information. [file 41598_2020_69939_MOESM1_ESM.pdf]

SUPPLEMENTARY DATA

Autophagy is a pro-survival adaptive response to heat shock in bovine cumulus-oocyte-complexes

Lais B. Latorraca†, Weber B. Feitosa†, Camila Mariano, Marcelo T. Moura, Patrícia K. Fontes, Marcelo F. G. Nogueira, Fabíola F. Paula-Lopes

†Authors contributed equally for this manuscript

Table S1. Results (least-squares means ± SEM) and p-value for interaction (temperature x autophagy inhibitor) of genes evaluated by RT-qPCR that did not present significant difference (P>0.05) between experimental groups.

FUNCTION	GENE	IVM-38.5ºC	3MA-38.5ºC	IVM-41ºC	3MA-41ºC	P value interaction
**Control of meiosis**	PDE5A	0.50 ± 0.09	0.60 ± 006	0.48 ± 0.06	0.57 ± 0.07	0.89
	NPR1	0.01 ± 0.002	0.01 ± 0.002	0.01 ± 0.002	0.01 ± 0.002	0.94
	NPR2	0.16 ± 0.04	0.16 ± 0.03	0.20 ± 0.03	0.19 ± 0.03	0.84
	NOS2	2.66 ± 0.73	2.52 ± 0.53	2.46 ± 0.53	3.51 ± 0.61	0.39
	NOS3	0.23 ± 0.05	0.25 ± 0.03	0.21 ± 0.03	0.22 ± 0.04	1.00
**Oocyte maturation**	EGFR	0.32 ± 0.04	0.31 ± 0.03	0.31 ± 0.03	0.28 ± 0.04	0.79
	GDF9	13.30 ± 2.46	11.36 ± 1.78	15.20 ± 1.78	14.49 ± 2.06	0.73
	H1FOO	9.86 ± 1.55	8.21 ± 1.13	10.55 ± 1.13	8.35 ± 1.30	1.00
	MAPK1	3.78 ± 0.75	3.36 ± 0.54	4.76 ± 0.54	3.53 ± 0.63	0.63
	OOSP1	26.92 ± 4.74	21.30 ± 3.43	28.73 ± 3.43	25.30 ± 3.79	0.68
**Oocyte competence to embryonic development**	DICER1	1.33 ± 0.44	1.35 ± 0.32	1.70 ± 0.32	1.91 ± 0.37	0.88
	HDAC2	0.85 ± 0.16	0.73 ± 0.12	0.83 ± 0.12	0.85 ± 0.14	0.59
	IGF1R	2.69 ± 0.65	2.52 ± 0.47	3.31 ± 0.47	3.06 ± 0.54	0.90
	IGFBP2	9.80 ± 1.75	8.97 ± 1.28	10.20 ± 1.28	8.13 ± 1.48	0.68
	VCAN	0.63 ± 0.11	0.43 ± 0.08	0.62 ± 0.08	0.59 ± 0.09	0.26
**Embryonic development**	POU5F1	3.31 ± 0.89	2.77 ± 0.64	3.55 ± 0.64	4.21 ± 0.75	0.57
	SOX2	1.23 ± 0.20	0.97 ± 0.14	1.25 ± 0.15	0.90 ± 0.17	0.79
**Cellular cycle**	CCND2	0.009 ± 0.004	0.004 ± 0.003	0.007 ± 0.002	0.01 ± 0.003	0.45
	CDCA8	27.5 ± 3.78	27.55 ± 2.74	32.99 ± 2.74	28.40 ± 3.17	0.52
	CDK6	0.04 ± 0.01	0.04 ± 0.009	0.03 ± 0.009	0.05 ± 0.01	0.56
**Cellular growth**	PA2G4	2.99 ± 0.89	2.68 ± 0.65	3.02 ± 0.65	4.03 ± 0.75	0.51
	STAT3	17.96 ± 3.63	17.79 ± 2.63	20.85 ± 2.63	23.96 ± 3.04	0.64
**Energy metabolism**	ATP5L	4.09 ± 0.71	3.24 ± 0.52	4.12 ± 0.52	3.52 ± 0.60	0.63
	GSK3A	4.84 ± 1.07	3.89 ± 0.78	4.79 ± 0.78	3.89 ± 0.9	0.98
	GFPT2	0.55 + 0.12	0.48 + 0.09	0.54 + 0.09	0.66 + 0.1	0.41
	GADPH	4.22 + 0.68	3.86 + 0.49	4.5 + 0.49	3.38 + 0.57	0.66
	PFKP	0.08 + 0.03	0.07 + 0.02	0.12 + 0.02	0.09 + 0.02	0.67
	PGK1	0.15 + 0.05	0.14 + 0.04	0.22 + 0.04	0.20 + 0.04	0.89
	PPARGC1A	1.29 + 0.36	1.15 + 0.26	1.40 + 0.26	1.74 + 0.3	0.64
**Lipid metabolism**	G6PD	1.25 + 0.25	1.01 + 0.18	1.31 + 0.18	1.20 + 0.21	0.76
	GPAM	0.12 + 0.04	0.12 + 0.03	0.14 + 0.03	0.17 + 0.03	0.73
	NR1H3	0.008 + 0.005	0.013 + 0.004	0.01 + 0.004	0.01 + 0.004	0.47
	PNPLA2	0.73 + 0.21	0.63 + 0.15	0.74 + 0.15	0.85 + 0.18	0.73

	PPARA	0.49 + 0.09	0.43 + 0.07	0.49 + 0.07	0.53 + 0.08	0.54
	SREBF1	0.22 + 0.05	0.22 + 0.03	0.24 + 0.03	0.17 + 0.04	0.43
	SREBF2	3.39 ± 0.51	3.48 ± 0.37	4.03 ± 0.37	2.82 ± 0.43	0.16
**DNA methylation**	DNMT1	8.47 + 1.17	7.36 + 0.86	8.77+ 0.86	7.09 + 0.99	0.78
	DNMT3A	1.27 + 0.41	1.24 + 0.3	1.74 + 0.3	1.89 + 0.35	0.71
	DNMT3B	6.47 + 1.01	6.07 + 0.73	7.45 + 0.73	6.55 + 0.85	0.75
	PAF1	4.90 + 1.36	4.38 + 0.98	6.45 + 0.98	6.81 + 1.14	0.87
**Transcriptor factor**	ATF4	0.96 + 0.14	0.91 + 0.11	0.8 + 0.11	0.64 + 0.13	0.49
	REST	0.14 + 0.02	0.14 + 0.02	0.13 + 0.02	0.10 + 0.02	0.58
**Apoptosis pathway**	BAX	1.66 + 0.28	1.39 + 0.2	1.83 + 0.2	1.52 + 0.23	0.91
	BCL2	0.04 + 0.01	0.03 + 0.008	0.04 + 0.008	0.03 + 0.009	0.94
	BID	0.20 + 0.04	0.21 + 0.03	0.19 + 0.03	0.24 + 0.04	0.84
	CASP3	0.11 + 0.01	0.09 + 0.007	0.08 + 0.007	0.08 + 0.008	0.19
	CASP9	0.37 + 0.07	0.30 + 0.05	0.35 + 0.05	0.39 + 0.06	0.45
	FOXO3	1.60 + 0.29	1.45 + 0.22	1.80 + 0.22	1.52 + 0.25	0.92
	NFKB2	1.45 + 0.27	1.49 + 0.20	1.67 + 0.20	1.45 + 0.23	0.57
	TNFRSF21	0.32 + 0.08	0.18 + 0.06	0.31 + 0.06	0.28 + 0.07	0.45
	TP53	0.11 + 0.04	0.09 + 0.03	0.13 + 0.03	0.15 + 0.03	0.98
**Cellular stress**	DDIT3	1.83 + 0.43	1.53 + 0.31	2.02 + 0.31	1.85 + 0.36	0.86
	HIF1A	0.68 + 0.08	0.74 + 0.06	0.77 + 0.06	0.67 + 0.07	0.26
	TFAM	0.04 + 0.01	0.03 + 0.01	0.05 + 0.01	0.05 + 0.01	0.38
	XBP1	2.04 + 0.58	1.87 + 0.42	2.40 + 0.42	2.75 + 0.48	0.50
**Heat shock response**	HSP5A	3.79 + 0.81	3.33 + 0.59	4.49 + 0.59	3.96 + 0.68	0.38
	HSPA1A	0.54 ± 0.1	0.56 ± 0.07	0.66 ± 0.07	0.44 ± 0.08	0.16
	HSP90AA1	19.87 + 3.81	17.28 + 2.76	23.68 + 2.76	19.66 + 3.19	0.84
	HSPD1	1.89 + 0.5	1.59 + 0.37	2.24 + 0.37	2.32 + 0.42	0.76
**Oxidative stress**	CAT	1.21 + 0.32	1.12 + 0.23	1.41 + 0.23	1.46 + 0.27	0.84
	GLRX2	0.15 + 0.06	0.11 + 0.04	0.16 + 0.04	0.19 + 0.05	0.77
	GPX1	0.30 + 0.07	0.33 + 0.05	0.35 + 0.05	0.39 + 0.06	0.87
	GPX4	0.66 + 0.12	0.73 + 0.09	0.74 + 0.09	0.60 + 0.10	0.34
	KEAP1	1.24 + 0.50	1.11 + 0.36	1.46 + 0.36	1.74 + 0.42	0.91
	NFE2L2	0.14 + 0.03	0.10 + 0.02	0.14 + 0.02	0.13 + 0.02	0.57
	PRDX1	8.40 + 2.85	8.36 + 2.06	9.81 + 2.06	12.41 + 2.39	0.80
	PRDX3	1.02 + 0.30	0.90 + 0.20	1.29 + 0.20	1.38 + 0.23	0.83
	SOD1	5.63 + 1.05	4.84 + 0.76	6.33 + 0.76	6.10 + 0.88	0.69
	SOD2	1.60 + 0.44	1.02 + 0.32	1.66 + 0.32	1.27 + 0.37	0.80
**Others**	PTGS2	0.58 + 0.10	0.51 + 0.07	0.61 + 0.07	0.55 + 0.08	0.93
	RGS2	27.84 + 6.56	24.27 + 4.76	31.50 + 4.76	34.32 + 5.50	0.60

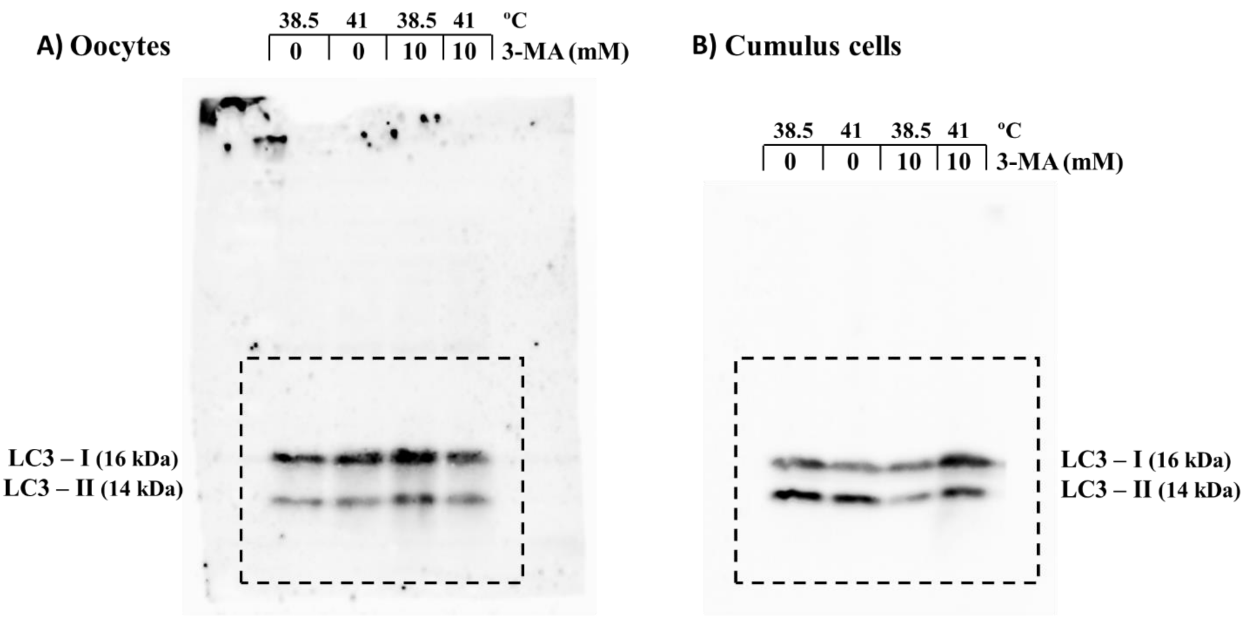

Figure S1: Uncropped blots of Figure 2 for oocytes (A) and cumulus cells (B). Images cropped to dotted-lines boxes. A single gel was used to show both types of LC3 (I and II).

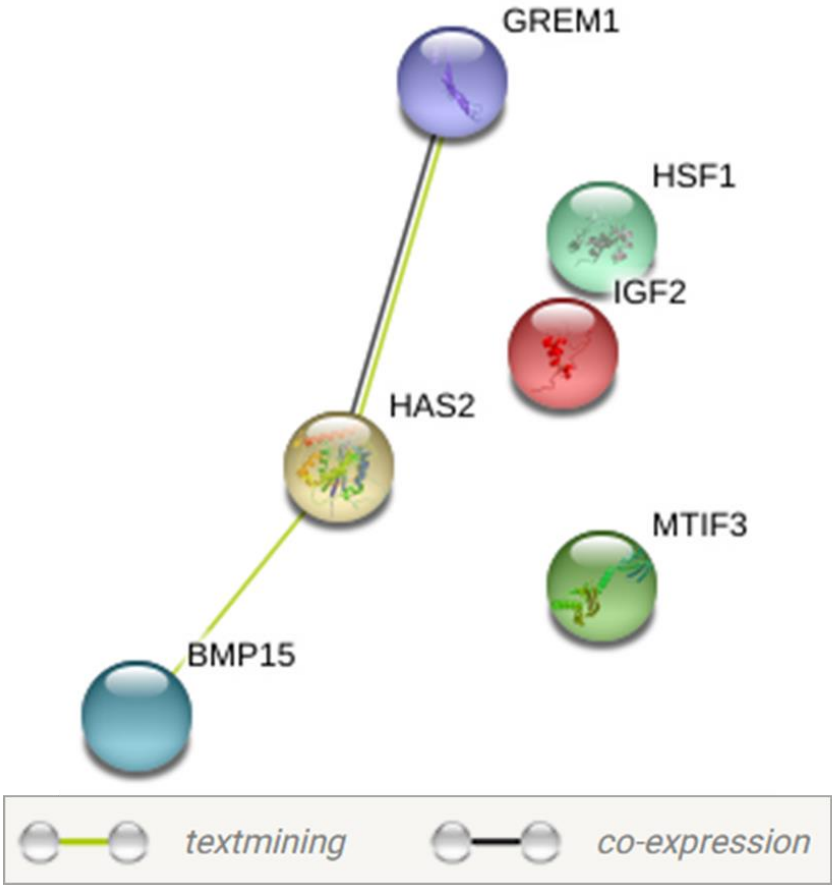

Figure S2. STRING network of protein-protein interactions between genes with significant difference ($P \leq 0.05$) of mRNA abundance among groups. Different line colors represent the relationship between the proteins translated from analyzed genes, as described in the legend.

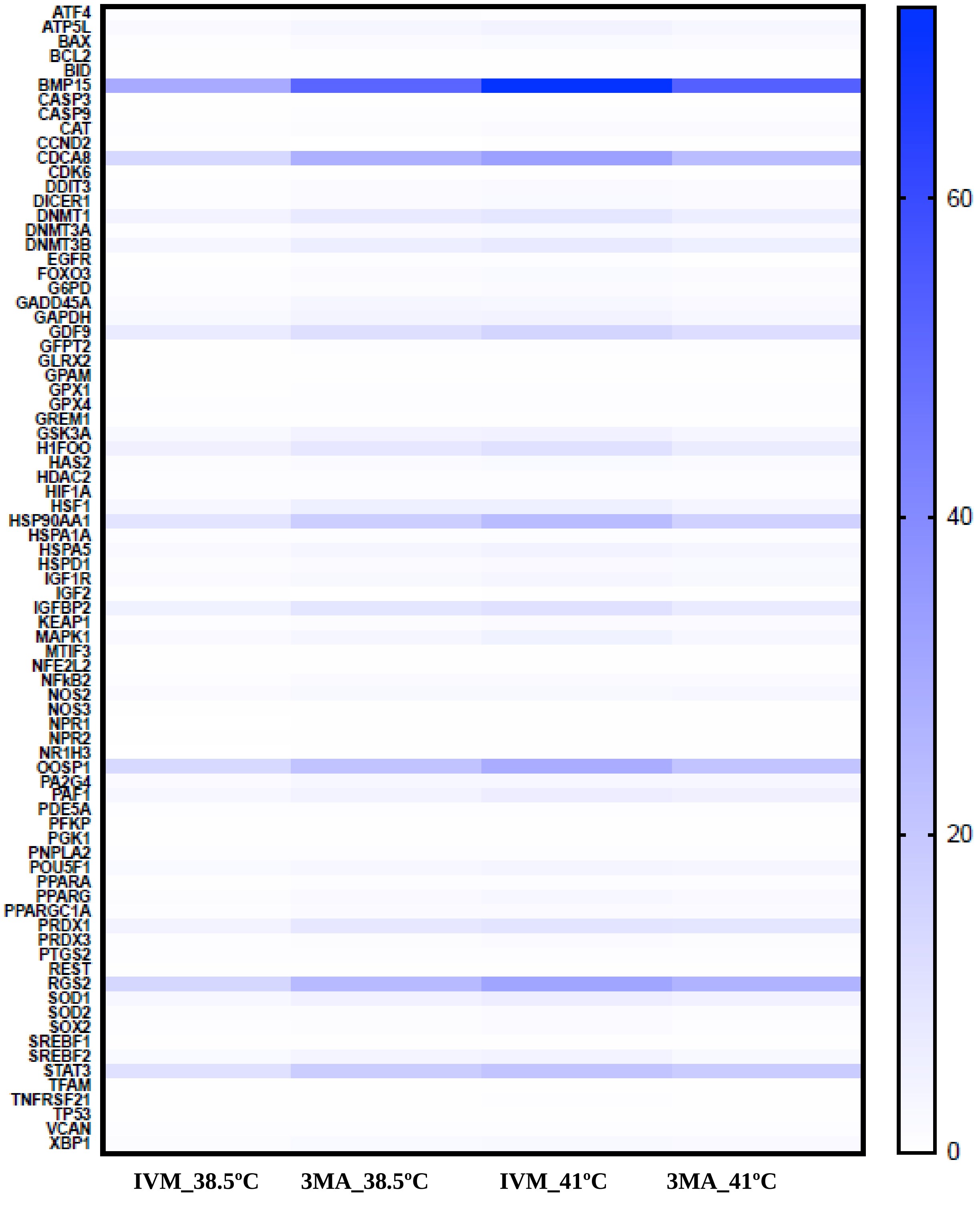

Figure S3. Heat map analysis of 2 fold change-ΔCq results obtained from gene expression analysis of bovine oocytes performed with Applied BiosystemsTaqMan Assays[83].
